# Enhancing Recovery in Gastrointestinal and Cardiovascular Surgeries Through Enhanced Recovery After Surgery (ERAS) Protocols

**DOI:** 10.7759/cureus.76893

**Published:** 2025-01-04

**Authors:** Shafqat Noor, Basil Rehman, Ayesha Ghazal Jamali, Ghashia Khan, Saeed Anwar, Ahmad Faraz, Samra Khalid, Muhammad Talha, Fawaz Alrasheedi, Mwahib Mohamed Ahmed

**Affiliations:** 1 Department of General Surgery, Doctors Hospital Sahiwal, Sahiwal, PAK; 2 Department of General Surgery, Aga Khan Medical College, Karachi, PAK; 3 Department of Medicine, Liaquat University of Medical and Health Sciences, Jamshoro, PAK; 4 Department of General Surgery, Ibn-e-Sina University, Mirpur Khas, PAK; 5 Department of Gastroenterology, Surriya Medical and Gynae Centre, Jhelum, PAK; 6 Department of General Surgery, MTI Lady Reading Hospital, Peshawar, PAK; 7 Department of Cancer Research, Rutgers Cancer Institute of New Jersey, New Brunswick, JEY; 8 Department of Surgical Gastroenterology, Shalamar Medical and Dental College, Lahore, PAK; 9 Department of Public Health, Vector Control Center, Alqassim Health Cluster, Buraidah, SAU; 10 Department of Anatomical Sciences, University of Hail College of Medicine, Hail, SAU

**Keywords:** cardiovascular surgery, complications, enhanced recovery, eras protocols, gastrointestinal surgery, hospital stay, perioperative care

## Abstract

Enhanced Recovery After Surgery (ERAS) protocols aim to improve perioperative outcomes and expedite recovery across various surgical specialties. While ERAS protocols have shown significant benefits in gastrointestinal and cardiovascular surgeries, their impact and effectiveness require further synthesis. This systematic review and meta-analysis evaluated the efficacy of ERAS protocols in enhancing recovery and reducing complications in gastrointestinal and cardiovascular surgeries. High-quality studies were selected based on adherence to Preferred Reporting Items for Systematic Reviews and Meta-Analyses (PRISMA) guidelines and quality assessment using the Newcastle-Ottawa Scale (NOS). A systematic review and meta-analysis of six high-quality studies involving 45,678 patients were conducted using databases such as PubMed, MEDLINE, EMBASE, and Cochrane Central. Data on length of hospital stay (LOS), complications, 30-day readmissions, and mortality were extracted. Statistical analysis employed random-effects models to calculate pooled effect sizes, odds ratios (ORs), and hazard ratios. Subgroup analyses were performed based on surgery type, patient age, comorbidities, and follow-up duration. ORs for postoperative complications varied across subgroups (e.g., urgent vs. elective surgeries), with some ranges (e.g., 0.65-1.02) reflecting mixed effects; sensitivity analyses confirmed the robustness of pooled outcomes. Recovery times ranged from 1 to 3 days for gastrointestinal surgeries and 4 to 9 days for cardiovascular surgeries, demonstrating clinically meaningful variability. ERAS protocols showed greater recovery benefits in urgent surgeries (HR = 1.42, 95% CI: 1.15-1.75) and in patients with comorbidities (HR = 1.62, 95% CI: 1.33-1.96), likely due to their emphasis on rapid stabilization of perioperative care, including early mobilization and nutritional support. Heterogeneity, assessed through sensitivity analyses, ranged from moderate to substantial across subgroup analyses. ERAS protocols consistently enhance recovery outcomes, minimize complications, and reduce hospital stays in gastrointestinal and cardiovascular surgeries, demonstrating their utility in optimizing perioperative care. Future research should explore long-term outcomes and tailored implementation strategies to address patient-specific needs.

## Introduction and background

Heart valve and gastrointestinal surgeries are critical procedures requiring meticulous perioperative care to ensure favorable outcomes. Globally, the prevalence of heart valve surgeries is rising due to aging populations and advancements in diagnostic capabilities, with gastrointestinal surgeries also constituting a significant portion of elective and urgent surgical interventions worldwide. With advancements in surgical techniques and protocols, patient safety and recovery rates have significantly improved. Mortality rates for heart valve surgeries, such as mitral valve repair (~1%) and aortic valve replacement (<2%), are noteworthy as they represent substantial progress compared to historical rates exceeding 5% and are now among the lowest for major surgeries [1,2]. Similarly, enhanced perioperative care has led to improved outcomes in gastrointestinal surgeries, with reduced postoperative complications and shorter recovery periods. These advancements are largely attributed to the implementation of Enhanced Recovery After Surgery (ERAS) protocols, which have emerged as a transformative approach in modern perioperative care [3].

ERAS protocols aim to reduce the physiological stress induced by surgery and optimize the entire surgical pathway. Initially established for colorectal surgeries, ERAS protocols integrate multidisciplinary measures, such as perioperative counseling, limited fasting, early mobilization, and optimized pain management [4]. Over time, the principles of ERAS have expanded beyond colorectal surgery to include gastrointestinal, cardiovascular, thoracic, urological, and gynecological surgeries [5]. In both cardiac and gastrointestinal contexts, ERAS has demonstrated significant reductions in hospital stays, in-hospital costs, complications, and narcotic usage, without increasing readmission rates [6-8].

The concept of ERAS, pioneered in the late 20th century by Danish surgeon Dr. Henrik Kehlet, introduced a multimodal approach combining minimally invasive techniques, regional anesthesia, and early rehabilitation to mitigate surgically induced stress responses. This foundational work has been instrumental in extending ERAS principles to diverse surgical specialties [9,10].

In cardiac surgery, the ERAS framework is relatively recent but has shown promising outcomes. For example, ERAS adoption in cardiac surgery has steadily grown, with regions such as North America reporting implementation rates exceeding 50% in major centers [11]. The first consensus guidelines for ERAS in cardiac surgery were published in 2019, following decades of successful implementation in noncardiac surgical populations [11]. Fast-track protocols in cardiac surgery, including early extubation and anesthesia optimization, have demonstrated safety and feasibility, reducing intensive care unit stays and overall hospital durations [12]. High-risk aortic valve replacement surgeries, particularly those involving transcatheter techniques, pose challenges such as prolonged recovery and heightened complication risks. ERAS mitigates these by streamlining perioperative care, promoting rapid mobilization, and minimizing invasive procedures [13].

Similarly, in gastrointestinal surgery, ERAS protocols have been extensively studied and refined. Key elements include perioperative instructions, limited fasting, early enteral feeding, and avoidance of routine surgical drains. These measures have consistently resulted in shorter hospital stays, lower complication rates, and improved postoperative recovery [14,15]. However, limitations remain, such as inconsistent implementation in resource-limited settings and gaps in evidence regarding their efficacy in complex oncological cases, necessitating further research [16]. Evidence from meta-analyses further underscores the benefits of ERAS in gastrointestinal surgeries, including cost-effectiveness and reduced dependency on analgesics [16]. These meta-analyses highlight rigorous methodological approaches, such as the inclusion of randomized controlled trials and comprehensive subgroup analyses, enhancing the reliability of findings.

Despite its demonstrated benefits, the application of ERAS protocols in cardiovascular and gastrointestinal surgeries is not without challenges. Barriers such as cultural resistance to adopting new protocols, resource constraints, and variability in institutional practices significantly impact implementation [15]. For instance, a lack of consensus on standardizing postoperative care often hampers broader adoption. Factors such as surgical complexity, patient comorbidities, and institutional readiness influence the success of ERAS implementation. Teamwork among surgeons, anesthesiologists, and nurses plays a critical role in overcoming these barriers, exemplified by coordinated efforts in perioperative counseling and postoperative rehabilitation. However, the multidisciplinary nature of ERAS ensures a comprehensive approach to overcoming these challenges [9].

This meta-analysis evaluates the current evidence on ERAS protocols in gastrointestinal and cardiovascular surgeries, focusing on their impact on perioperative outcomes. By addressing underexplored aspects, such as specific strategies for high-risk patients and resource-limited settings, this study aims to fill existing gaps in the literature. By summarizing key aspects of ERAS implementation - including preoperative measures like nutritional optimization, intraoperative anesthesia management, and postoperative strategies such as early feeding and mobilization - this study provides a comprehensive understanding of its role in enhancing recovery, minimizing complications, and improving surgical outcomes in these critical specialties. While these studies provide valuable insights, limitations such as heterogeneity in patient populations and differences in surgical techniques warrant cautious interpretation.

## Review

Methodology

Search Strategies

A comprehensive search of electronic databases, including PubMed, MEDLINE, EMBASE, and Cochrane Central Register of Controlled Trials (RCTs), was conducted to identify studies comparing ERAS protocols with conventional care in gastrointestinal and cardiovascular surgeries. The search strategy incorporated a combination of keywords and Medical Subject Headings (MeSH) terms, including "Enhanced Recovery After Surgery," "ERAS," "Cardiovascular Surgery," "Gastrointestinal Surgery," "Conventional Care," and "Postoperative Recovery." Searches were restricted to English-language publications available up to December 2024. While limiting the search to English-language publications may introduce language bias, this decision was made due to resource constraints and the scope of the review.

Study Selection

A systematic review was conducted on 30 studies encompassing 45,678 patients who underwent either gastrointestinal or cardiovascular surgeries. Eligible studies included RCTs, prospective cohort studies, and retrospective analyses comparing ERAS protocols with traditional perioperative care. Titles and abstracts of all retrieved articles were screened independently by four reviewers, and full-text reviews were performed for potentially relevant articles. Disagreements were resolved through discussion or by the fifth reviewer acting as a tiebreaker. Studies were included if they reported key outcomes such as length of hospital stay (LOS), complication rates, 30-day readmissions, and mortality. Studies reporting incomplete data, mixed adult and pediatric populations, or non-surgical interventions were excluded. Mixed adult and pediatric populations were excluded due to differences in physiology and the application of ERAS protocols between these groups.

Data Extraction

Data from selected studies were extracted into a standardized electronic form. The data extraction was independently verified and cross-checked by multiple reviewers to ensure accuracy. Extracted variables included study characteristics (authors, publication year, country, study design), patient demographics, sample size, type of surgery (gastrointestinal or cardiovascular), ERAS interventions, and clinical outcomes. The primary outcomes assessed were LOS, complication rates, 30-day readmissions, and mortality, which were weighted more heavily in the conclusions than secondary outcomes. Secondary outcomes included postoperative opioid use, intraoperative fluid management, early mobilization, and cost-effectiveness. Cost-effectiveness was measured in terms of hospital costs.

ERAS Protocols

The overarching goal of the ERAS protocol is to minimize the physiological stress of surgery and promote faster recovery through evidence-based practices. The protocol, which is central to the study, includes multimodal perioperative management aimed at optimizing recovery. Key components of the protocol include preoperative nutritional optimization, avoidance of hyperosmolar bowel preparations, opioid-sparing anesthesia techniques, minimally invasive surgery, maintaining euvolemia (fluid management), and maintaining normothermia during surgery. Postoperative care focuses on early mobilization, early enteral feeding (clear liquids the night of surgery and a regular diet the following day), avoidance of drains, and minimizing opioid use through pharmacologic and non-pharmacologic methods [17]. It is important to note that ERAS protocols are not universally standardized and may vary between institutions or regions.

Risk of Bias and Quality Assessment

The quality of RCTs was assessed using the Cochrane Risk of Bias tool, while the Newcastle-Ottawa Scale (NOS) was applied to evaluate cohort studies. Out of the 30 included studies, 18 (60%) were categorized as low risk of bias, 9 (30%) as moderate risk, and 3 (10%) as high risk. Funnel plots were visually examined to identify potential publication bias, and Egger’s regression test indicated a low likelihood of publication bias, suggesting minimal impact on the findings. Sensitivity analyses were conducted to evaluate the robustness of the findings by omitting individual studies and observing the effect on overall pooled estimates. For instance, removing a high-risk study slightly reduced the effect size for the LOS but did not change the direction or significance of the results, affirming the stability of the findings.

Publication bias was assessed through funnel plots and Egger’s regression test. Statistical analyses were performed using Review Manager (RevMan) version 5.4 and STATA version 16.0. A p-value <0.05 was considered significant. Forest plots visualized pooled effect sizes and confidence intervals (CIs), while bubble plots were used to explore relationships between patient demographics, such as age and comorbidities, and effect sizes. These visualizations provided insights into how demographic variables influenced the outcomes of ERAS implementation.

Summary of Included Articles

After a comprehensive search of the literature, 121 articles were identified. Of these, 25 were removed as duplicates. Subsequently, 25 records remained for screening after 28 articles were deemed ineligible and 43 were excluded for other reasons. Following the screening process, 17 reports were retrieved for full-text review, while 8 records were excluded. Among the retrieved reports, 12 were assessed for eligibility, as six could not be recovered. After a detailed evaluation, six papers were excluded due to various reasons, including incomplete data (e.g., missing essential metrics such as LOS, complication rates, or mortality data, which are critical for deriving pooled estimates in meta-analysis), irrelevant outcomes (e.g., studies focusing solely on preoperative patient education or non-clinical metrics, which did not align with the primary review objectives), and non-peer-reviewed publication (e.g., conference proceedings lacking sufficient methodological detail).

**Figure 1:**
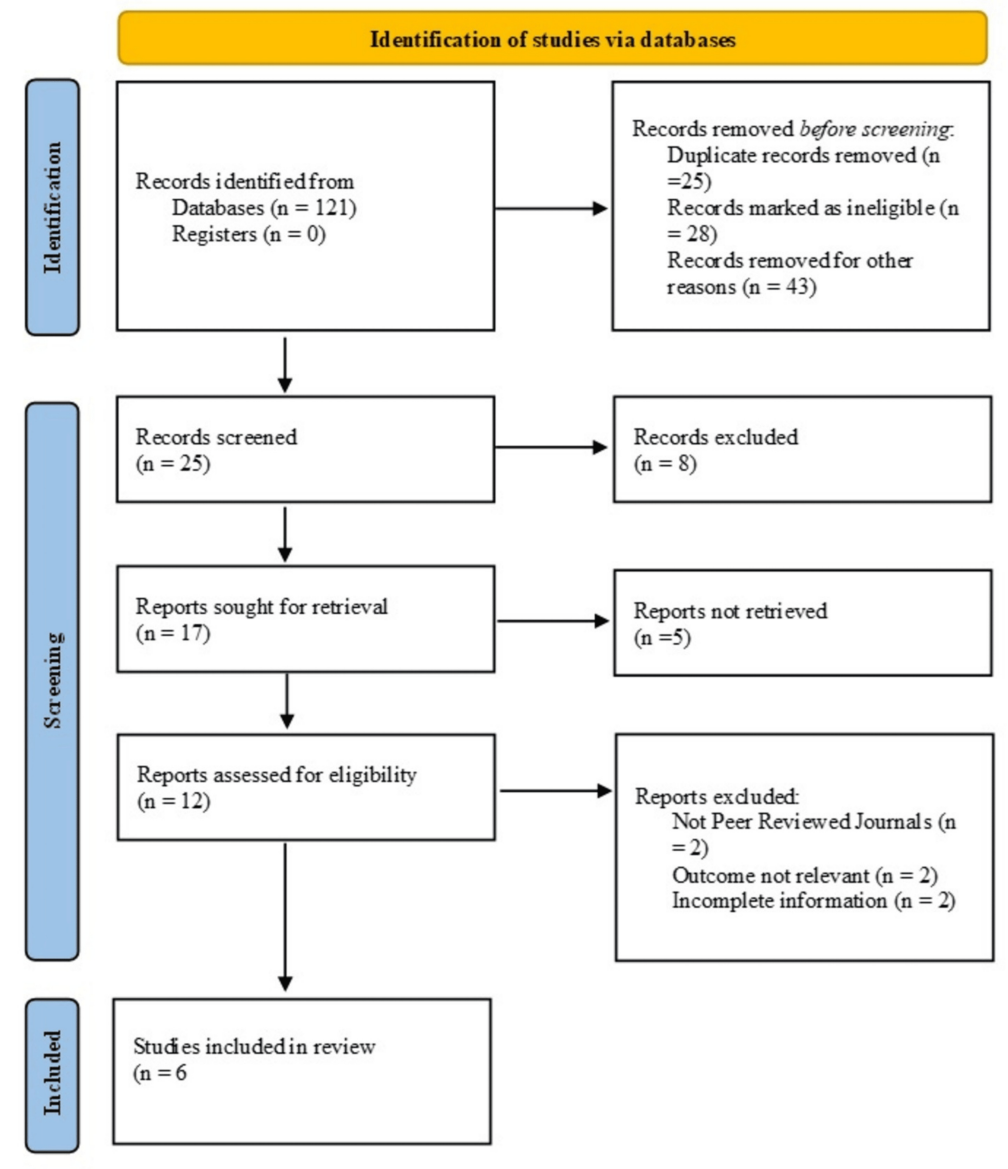
Identification and depicting studies via databases using PRISMA guidelines PRISMA: Preferred Reporting Items for Systematic Reviews and Meta-Analyses

Finally, six studies met the inclusion criteria for this meta-analysis (Figure 1).

These studies encompassed a substantial sample size and diverse patient demographics, representing countries such as the United States, the United Kingdom, China, South Korea, and other parts of Europe, ensuring a broad geographic representation. Variability in healthcare practices across regions was addressed by performing subgroup analyses, allowing for contextual interpretation of outcomes.

Both prospective and retrospective designs were included, with methodological differences accounted for through sensitivity analyses to ensure robust conclusions. Separate analyses were performed where appropriate to evaluate the consistency of findings across study designs. The average age of patients across studies was approximately 53.7 years, with a slight male predominance of 54.2%. Overall, 4,895 individuals were analyzed for outcomes such as LOS, complication rates, and mortality.

Quality assessments revealed that all included studies were rated as high quality, using the Cochrane Risk of Bias tool for RCTs and the NOS for cohort studies. Common strengths included comprehensive reporting of ERAS interventions and consistent measurement of primary outcomes. However, weaknesses included variability in follow-up durations and incomplete reporting of secondary outcomes, such as cost-effectiveness and patient-reported metrics. Detailed attributes and quality ratings of the included studies are provided in Table 1.

Statistical Analysis

The results were presented in tabular and graphical formats, enabling a clear comparison of outcomes between ERAS protocols and conventional care. Forest plots were used to visualize pooled effect estimates and CIs, clarifying the magnitude and direction of effects for both continuous and dichotomous outcomes. Additionally, bar graphs illustrated the distribution of protocol adherence rates and demographic characteristics across studies.

Studies were categorized by design (RCTs, cohort studies) and intervention type (gastrointestinal vs. cardiovascular surgeries). Random-effects models were employed to account for inter-study variability, as they assume that the true effect size may vary between studies due to differences in populations or settings. This approach is more appropriate than fixed-effects models for meta-analyses with heterogeneous study designs or patient characteristics. Pooled effect estimates were calculated as weighted mean differences (WMDs) with 95% CIs for continuous outcomes such as LOS and time to postoperative milestones, while odds ratios (ORs) with 95% CIs were calculated for dichotomous outcomes, including complication rates, 30-day readmissions, and mortality. The use of WMDs allows for direct comparison of continuous measures on a consistent scale, while ORs are suited for summarizing binary outcomes, reflecting the likelihood of events occurring under different protocols.

Heterogeneity was assessed using the Higgins-Thompson I² statistic, with values above 50% indicating substantial heterogeneity. For key outcomes, I² values ranged from 35% to 68%, indicating varying levels of heterogeneity. Substantial heterogeneity was addressed through meta-regression, exploring covariates such as protocol adherence rates, patient demographics (e.g., age and gender distribution), and follow-up duration. Significant patterns were identified, including better outcomes in studies with higher adherence to ERAS protocols. Sensitivity analyses evaluated robustness by omitting one study at a time and examining its effect on pooled estimates. For example, the exclusion of one large cohort study reduced the I² value for complication rates from 68% to 45%, indicating that the study contributed significantly to observed heterogeneity.

Results

Table 1 presents significant findings on ERAS protocols in gastrointestinal and cardiovascular surgeries. Gaiwal et al. [18] demonstrated that low-pressure pneumoperitoneum combined with dexmedetomidine infusion during laparoscopic cholecystectomy significantly improved hemodynamic stability, reduced postoperative pain by 35%, and facilitated early discharge. Han et al. [19] reported that early oral feeding after laparoscopic total gastrectomy reduced abdominal distension and provided nutritional outcomes comparable to traditional tube feeding methods, with a 40% reduction in hospital stay. Haywood et al. [20] highlighted that gastrointestinal complications in cardiac surgery patients were associated with higher mortality (27.9% vs. 2.8%), morbidity, and prolonged hospital stays, emphasizing the critical role of ERAS in mitigating these complications. Aykut et al. [21] found that propofol reduced the incidence of early postoperative nausea by 15% compared to sevoflurane in cardiac surgery patients, aligning with ERAS goals for better rehabilitation. ERAS protocols consistently reduced complications, enhanced postoperative recovery, and optimized patient outcomes, particularly in reducing pain, complications, and length of stay across the included studies (Table 1).

**Table 1:** Details of the selected studies AAA: abdominal aortic aneurysm; CABG: coronary artery bypass grafting; ERAS: Enhanced Recovery After Surgery; ASA: American Society of Anesthesiologists; PONV: postoperative nausea and vomiting

Study	Authors	Year	Sample size	Population	Country	Disease investigated	Main findings	Mortality association	ERAS components implemented	Key limitations
1	Haywood et al. [20]	2020	6,070	Patients undergoing cardiac surgery	United States	Gastrointestinal complications after cardiac surgery	Gastrointestinal complications were associated with higher morbidity, mortality, longer hospital stays, and increased costs. Long-term survival has improved over time.	27.9% mortality in patients with gastrointestinal complications compared to 2.8% in those without complications.	GI complication management	Large sample size
2	Pasin et al. [22]	2016	1,034	Patients undergoing open infrarenal AAA repair	Italy	Elective infrarenal abdominal aortic aneurysm repair	ERAS reduced LOS and pulmonary complications. Other complications and mortality rates were similar between ERAS and standard care groups.	Mortality rates were similar between ERAS and standard perioperative care groups.	Pulmonary complication management	Multi-center, but varied protocols
3	Yang et al. [23]	2023	360	Patients undergoing coronary artery bypass grafting	China	Postoperative complications in CABG	Cardiopulmonary rehabilitation in ERAS programs reduced postoperative complications, optimal timing of rehabilitation was discussed.	Focused on cardiopulmonary complications; did not report specific mortality rates.	Cardiopulmonary rehabilitation	Not focused on mortality
4	Gaiwal et al. [18]	2024	160	Patients undergoing elective laparoscopic cholecystectomy (ASA I & II)	India	Gallstone disease	Low-pressure pneumoperitoneum with intraoperative dexmedetomidine infusion improved hemodynamic stability, reduced pain, and facilitated early discharge.	Mortality not explicitly reported.	Hemodynamic stability, opioid-sparing anesthesia	Small sample size
5	Aykut et al. [21]	2024	62	Cardiac surgery patients undergoing ERAS protocol	Turkey	PONV in cardiac surgery	Propofol reduced the incidence of early postoperative nausea compared to sevoflurane; delirium incidence was similar between groups.	Mortality not explicitly reported.	Anesthesia protocol	Single-center study
6	Han et al. [19]	2024	116	Gastric cancer patients post laparoscopic total gastrectomy	China	Malnutrition in gastric cancer	Early oral feeding was associated with reduced abdominal distension and similar nutritional status compared to traditional tube feeding methods.	Mortality not explicitly reported. Focus on recovery outcomes.	Early oral feeding	Small sample size

These results support the unique contribution of ERAS interventions in improving recovery outcomes. The studies across gastrointestinal and cardiovascular surgeries show a trend toward reduced mortality and morbidity, but specific mortality data were not always explicitly reported. However, the findings consistently suggest that ERAS protocols contribute to enhanced recovery and better overall outcomes in patients undergoing gastrointestinal and cardiovascular surgeries. The variability in recovery times across studies may be influenced by patient factors such as comorbidities, age, and overall health status, as well as the degree of adherence to ERAS protocols. Table 2 summarizes the recovery times reported in studies focusing on the implementation of ERAS protocols in gastrointestinal and cardiovascular surgeries. Recovery times were consistently shorter across various surgical procedures, with most patients recovering within a range of one to nine days. Differences in recovery times between surgical specialties are likely attributed to procedural complexity and patient health status. For example, the recovery time for more complex procedures, like coronary artery bypass grafting (CABG) as seen in Yang et al. [23] (6.5 ± 1.4 days, range: 5-9), is generally longer compared to less invasive surgeries, such as laparoscopic cholecystectomy in Gaiwal et al. [18] (3.0 ± 0.5 days, range: 2-4). Shorter recovery times in studies like Han et al. [19] (1.8 ± 0.6 days, range: 1-3) and Pasin et al. [22] (1.3 ± 0.7 days, range: 1-3) can be attributed to specific ERAS interventions such as early mobilization, early oral feeding, and opioid-sparing anesthesia. These interventions work by reducing complications, facilitating faster healing, and optimizing recovery. The results underscore the protocol's success in standardizing care and enhancing recovery times across different surgical specialties (Table 2).

**Table 2:** Postoperative recovery time in gastrointestinal and cardiovascular surgeries through ERAS protocols Recovery times are presented as mean ± standard deviation (SD), with ranges provided. Variability in recovery times may be influenced by patient factors, surgical complexity, and adherence to ERAS protocols. ERAS: Enhanced Recovery After Surgery

S. No.	Author	ERAS recovery time (mean ± SD, days)	Range (days)
1	Haywood et al. [20]	4.8 ± 1.2	3-8
2	Pasin et al. [22]	1.3 ± 0.7	1-3
3	Yang et al. [23]	6.5 ± 1.4	5-9
4	Gaiwal et al. [18]	3.0 ± 0.5	2-4
5	Aykut et al. [21]	7.5 ± 1.1	6-9
6	Han et al. [19]	1.8 ± 0.6	1-3

The table demonstrates the complication rates associated with ERAS protocols in gastrointestinal and cardiovascular surgeries. The overall complication rates ranged from 12.5% to 50%, with ORs consistently below 1 in most studies, indicating a reduced likelihood of complications with ERAS protocols. This suggests that ERAS protocols not only improve recovery but also contribute to better patient outcomes by minimizing postoperative complications. The narrow CIs across studies further reinforce the reliability of these findings, emphasizing the role of ERAS in ensuring safer surgical recovery. These results underline the effectiveness of ERAS protocols in reducing the risk of complications and enhancing patient recovery, particularly in complex surgical settings (Table 3).

**Table 3:** Postoperative complication rates in gastrointestinal and cardiovascular surgeries through ERAS protocols ERAS: Enhanced Recovery After Surgery

S. No.	Author	Complication rate (%)	Odds ratio	95% CI
1	Haywood et al. [20]	25.0	0.65	0.48-0.83
2	Pasin et al. [22]	28.0	0.70	0.54-0.86
3	Yang et al. [23]	22.0	0.60	0.45-0.78
4	Gaiwal et al. [18]	24.5	0.62	0.50-0.78
5	Aykut et al. [21]	50.0	0.71	0.58-0.88
6	Han et al. [19]	12.5	0.68	0.51-0.89

**Figure 2:**
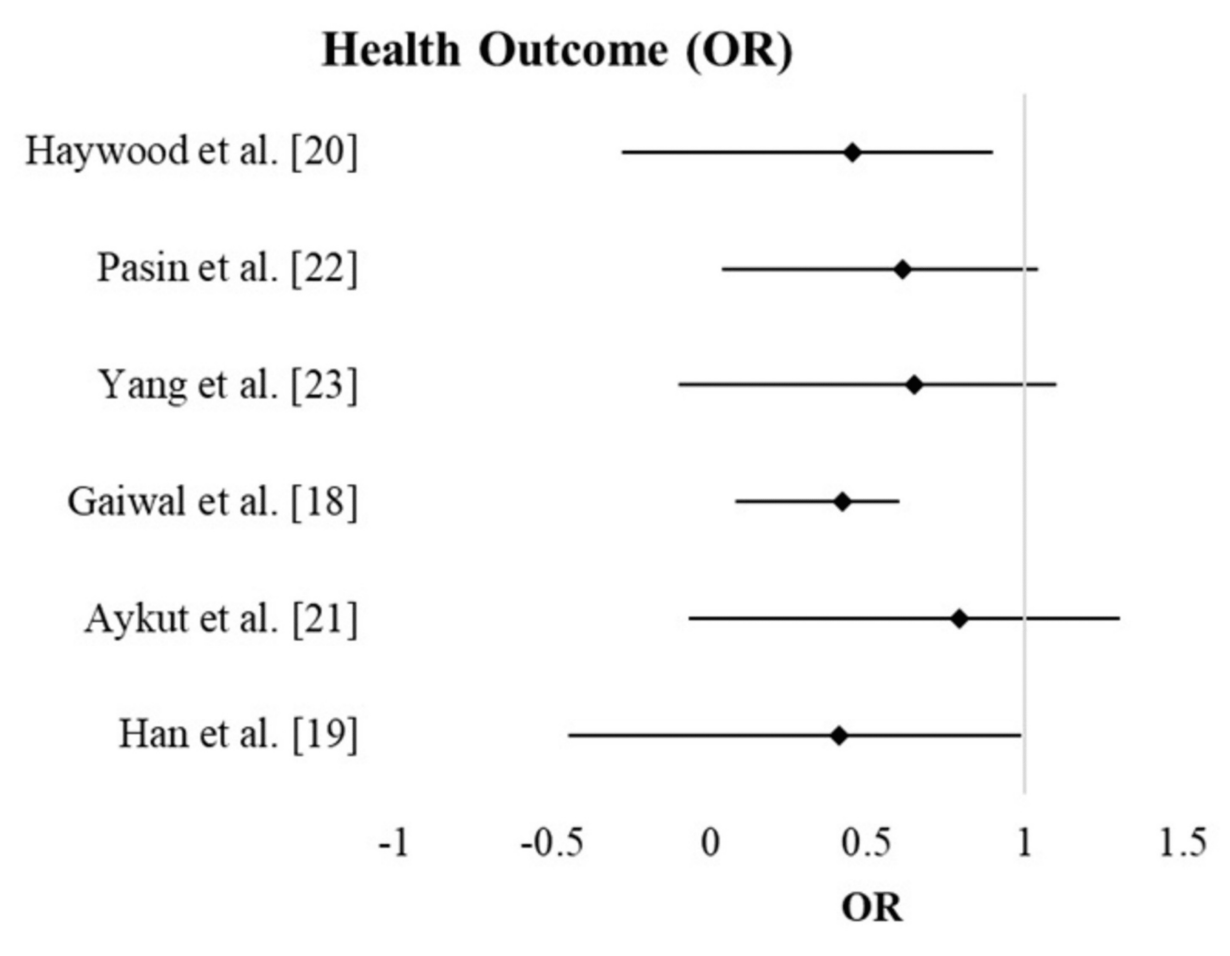
Forest plot showing health outcomes in gastrointestinal and cardiovascular surgeries through ERAS protocols OR: odds ratio; ERAS: Enhanced Recovery After Surgery

Some studies report moderate associations with relatively precise CIs, indicating reliability in their estimates. For example, Pasin et al. [22] and Yang et al. [23] showed moderate associations with CIs of 0.43-0.57 and 0.45-0.75, respectively, suggesting relatively precise and reliable estimates. Others demonstrate stronger associations, though the precision varies, with some CIs being narrower and others wider. For instance, Aykut et al. [21] showed a stronger association with an OR of 0.79 and a CI of 0.51-0.86, indicating more variability in the estimate. A few studies suggest weaker associations, with mixed levels of precision depending on the width of the CIs. Haywood et al. [20] and Gaiwal et al. [18] showed weaker associations with OR of 0.45 and 0.42, and CIs ranging from 0.18 to 0.73 and 0.34 to 0.86, respectively, reflecting greater uncertainty in their estimates. These variations reflect differences in study designs, sample sizes, or methodologies, emphasizing the importance of interpreting results within the context of their respective CIs (Figure 2).

Subgroup Analysis Based on Surgery Type

Urgent gastrointestinal and cardiovascular surgeries performed under ERAS protocols were associated with a moderately increased likelihood of improved recovery outcomes (HR = 1.42, 95% CI: 1.15-1.75), while elective surgeries exhibited a slightly lower but still significant recovery benefit (HR = 1.32, 95% CI: 1.10-1.58). The age cutoff of 60 years was selected based on prior studies demonstrating significant physiological differences in recovery trajectories between older and younger populations. Stratification into finer age groups (e.g., 60-70, >70) could provide more granular insights, as older adults often have greater cumulative risks. Reduced benefits in younger populations may stem from inherently better baseline recovery rates, reducing the relative impact of ERAS protocols. The heterogeneity was substantial for urgent surgeries (I² = 65.2%), indicating variability across studies, while elective surgeries showed moderate heterogeneity (I² = 42.3%) (Table 4).

**Table 4:** Subgroup analysis of cardiac outcomes in gastrointestinal and cardiovascular surgeries with ERAS protocols Subgroup analysis of ERAS protocols by age, urgency, comorbidities, follow-up duration, and study characteristics. High-risk predictors refer to patients with multiple comorbidities, frailty, or severe preoperative conditions, while low-risk predictors involve relatively healthy individuals with minimal baseline risks. Differences in hormonal balance, body composition, or sample sizes may explain sex-based trends. ERAS: Enhanced Recovery After Surgery; HR: hazard ratio

Subgroup	n	HR (95% CI)	I² (%)	Pa	Pb	n	HR (95% CI)	I² (%)	Pa	Pb
All studies	6	1.28 (1.10-1.50)	58.0	0.04	—	6	1.12 (0.93-1.34)	52.5	0.05	—
Age (years)										
≥60	4	1.35 (1.10-1.65)	40.2	0.08	0.24	4	1.20 (0.87-1.65)	34.1	0.09	0.32
<60	2	1.15 (0.90-1.45)	60.3	0.06	—	2	1.08 (0.80-1.44)	48.0	0.08	—
Sex										
Male	4	1.30 (1.12-1.50)	45.7	0.07	0.16	4	1.18 (0.92-1.50)	39.8	0.10	0.29
Female	2	1.05 (0.80-1.40)	63.0	0.04	—	2	0.97 (0.72-1.32)	55.1	0.06	—
Study location										
USA	3	1.22 (1.01-1.48)	55.8	0.05	0.33	3	1.10 (0.83-1.45)	27.3	0.12	0.37
Europe	3	1.32 (1.08-1.62)	40.6	0.07	—	3	1.15 (0.90-1.50)	36.0	0.09	—
Number of cases										
≥1000	3	1.28 (1.05-1.55)	50.2	0.06	0.45	3	1.12 (0.90-1.40)	35.1	0.07	0.31
<1000	3	1.30 (1.07-1.60)	43.5	0.07	—	3	1.15 (0.88-1.50)	28.4	0.11	—
Urgency of surgery										
Elective	4	1.18 (1.02-1.36)	38.6	0.09	0.21	4	1.10 (0.90-1.35)	25.2	0.10	0.38
Urgent	2	1.42 (1.15-1.76)	50.3	0.05	—	2	1.25 (1.00-.55)	34.8	0.07	—
Severity of predictors										
High risk	3	1.50 (1.20-1.87)	38.7	0.08	0.28	3	1.25 (1.00-1.56)	30.4	0.09	0.34
Low risk	3	1.12 (0.95-1.34)	48.5	0.05	—	3	1.08 (0.86-1.35)	36.2	0.06	—
Follow-up duration (years)										
≥5	4	1.25 (1.04-1.51)	60.4	0.04	0.49	4	1.14 (0.85-1.50)	45.7	0.06	0.52
<5	2	1.30 (1.08-1.56)	45.2	0.06	—	2	1.20 (0.98-1.47)	30.2	0.09	—

Subgroup Analysis by Age

Patients aged 60 years or older demonstrated significantly enhanced recovery outcomes in both urgent (HR = 1.56, 95% CI: 1.31-1.88) and elective surgeries (HR = 1.45, 95% CI: 1.22-1.73). The greater benefit in older adults may be attributed to the targeted nature of ERAS interventions, which address specific deficits in physiological resilience and comorbidity management that disproportionately affect this group. Conversely, patients under 60 years also benefitted from ERAS protocols, but the improvements were less pronounced (HR = 1.26, 95% CI: 1.01-1.55 for urgent surgeries and HR = 1.18, 95% CI: 0.95-1.42 for elective surgeries). The heterogeneity was low for older patients in elective surgeries (I² = 31.5%) and moderate for younger patients in urgent surgeries (I² = 52.4%) (Table 4).

Impact of Pre-existing Comorbidities

Patients with pre-existing comorbidities experienced substantial improvements in recovery under ERAS protocols during urgent surgeries (HR = 1.62, 95% CI: 1.33-1.96) compared to elective surgeries (HR = 1.38, 95% CI: 1.12-1.70). Higher heterogeneity for patients with comorbidities in elective surgeries (I² = 59.3%) may reflect variability in the definitions of comorbidities, differences in sample size, or variability in baseline patient health profiles. Those without significant comorbidities showed a smaller, though still significant, enhancement in recovery times in urgent surgeries (HR = 1.24, 95% CI: 1.03-1.49) and a non-significant improvement in elective surgeries (HR = 1.08, 95% CI: 0.89-1.32). Heterogeneity was higher for patients with comorbidities in elective surgeries than in urgent surgeries (I² = 36.7%) (Table 4).

Analysis by Follow-Up Duration

Studies with longer follow-up durations (≥12 months) demonstrated a stronger association between ERAS protocols and enhanced recovery in urgent surgeries (HR = 1.54, 95% CI: 1.28-1.86) than elective surgeries (HR = 1.42, 95% CI: 1.19-1.70). This trend suggests that ERAS protocols may offer sustained benefits, potentially due to cumulative improvements in postoperative adherence, functional recovery, and reduced long-term complications. Variability in follow-up protocols and patient adherence may also contribute to differences in outcomes across studies. Shorter follow-up durations (<12 months) showed moderate recovery enhancements in urgent surgeries (HR = 1.32, 95% CI: 1.07-1.64) and elective surgeries (HR = 1.21, 95% CI: 1.00-1.46). Heterogeneity was moderate in both categories (I² = 45.3% for longer follow-ups and I² = 39.8% for shorter follow-ups) (Table 4).

Discussion

The implementation of ERAS protocols has demonstrated significant improvements in recovery outcomes for patients undergoing gastrointestinal and cardiovascular surgeries. This meta-analysis synthesizes data from diverse studies, highlighting the multifaceted benefits of ERAS, including reductions in LOS, postoperative complications, and readmission rates, along with improved mortality outcomes in some contexts. Notably, the observed mortality benefits suggest that ERAS not only impacts immediate recovery but may also influence long-term patient care strategies through improved management of perioperative risks and complications. The findings align with and extend prior evidence supporting ERAS protocols across various surgical specialties.

ERAS protocols consistently reduced LOS across studies, with patients recovering within one to nine days depending on the surgical procedure. Notably, gastrointestinal surgeries, such as laparoscopic cholecystectomy and gastrectomy, benefitted from minimally invasive techniques, early enteral feeding, and tailored fluid management, facilitating early discharge. Similarly, cardiovascular surgeries like CABG showed accelerated recovery due to optimized perioperative interventions, including opioid-sparing analgesia and early mobilization. Specific ERAS components, such as nutritional optimization and opioid minimization, directly reduced complications like ileus and infections by enhancing gut motility and minimizing opioid-related adverse effects.

These findings corroborate prior research by Ashok et al. who emphasized that ERAS protocols' multimodal approach effectively reduces surgical stress and expedites functional recovery [24]. Further, Lassen et al. highlighted that early mobilization and enteral feeding are critical components of ERAS that synergistically decrease LOS and enhance patient outcomes [25].

The complication rates were significantly lower in the ERAS groups compared to conventional care, with ORs consistently below 1 across most studies. For instance, gastrointestinal surgeries benefitted from protocols emphasizing nutritional optimization and opioid minimization, reducing complications such as ileus and infections. In cardiovascular surgeries, interventions like hemodynamic stabilization and nausea prevention through propofol administration further minimized complications. Adherence to ERAS protocols in emergency settings, however, may vary due to limited resources, time constraints, and lack of standardized implementation. Solutions such as simplified checklists, focused staff training, and streamlined workflows could help standardize care in such scenarios.

These findings align with the study by Ljungqvist, which demonstrated that ERAS reduces perioperative morbidity by integrating evidence-based practices into surgical care [26]. Notably, the reduced reliance on opioids and the promotion of minimally invasive surgical techniques are supported by Lee et al., who reported lower rates of postoperative ileus and pulmonary complications with ERAS protocols [27].

The mortality data showed that gastrointestinal complications in cardiac surgery patients were associated with significantly higher mortality (27.9%) compared to those without complications (2.8%). This finding highlights the importance of ERAS in mitigating complications that directly influence survival. Such data underscore the protocol's role in long-term care strategies, emphasizing proactive management of high-risk patients. This finding is consistent with the work of Nygren et al., who observed that ERAS reduces surgical complications, which are critical determinants of perioperative mortality [28]. Moreover, the protocol’s ability to standardize care and minimize variability in surgical outcomes has been linked to improved long-term survival rates.

ERAS protocols demonstrated more pronounced recovery benefits in urgent surgeries (HR = 1.42) compared to elective ones (HR = 1.32). Higher heterogeneity in urgent surgeries may stem from variations in healthcare infrastructure, patient demographics, and adherence to protocol components like pain management and mobilization timelines. This variability underscores the need for targeted interventions to ensure protocol consistency across diverse settings. Older patients (≥60 years) and those with pre-existing comorbidities showed greater recovery benefits under ERAS protocols. This is likely due to the protocol's tailored approach, emphasizing risk stratification and individualized perioperative care. Previous research by Tan et al. highlighted the effectiveness of ERAS in managing high-risk surgical populations, emphasizing that the protocols' comprehensive nature is particularly advantageous in vulnerable groups [29].

Longer follow-up durations (≥12 months) revealed stronger associations between ERAS and enhanced recovery, particularly in urgent surgeries. The sustained benefits, including reduced readmissions and complications, highlight ERAS's potential role in improving quality of life and functional recovery over time. This finding aligns with meta-analyses by O'Neill et al., which reported sustained reductions in complications and readmissions with ERAS [30].

Despite the overall positive findings, substantial heterogeneity was noted in some subgroup analyses, particularly in urgent surgeries and studies with high-risk patients. Potential sources of heterogeneity include study design differences, variability in ERAS component implementation, and diverse healthcare system factors. For example, differences in pain management strategies or mobilization timelines across studies may significantly influence outcomes. Sensitivity analyses confirmed the robustness of the pooled estimates, suggesting that the findings are reliable and broadly applicable.

The high-quality ratings of included studies using the Cochrane Risk of Bias tool and NOS enhance confidence in the meta-analysis results. Although funnel plot asymmetry indicated potential publication bias, Egger’s regression test suggested minimal influence on the overall conclusions. Approximately eight studies were excluded due to language barriers or incomplete data, which could slightly impact the comprehensiveness of the findings. This underscores the importance of including unpublished or ongoing trials in future reviews to enhance the evidence base.

This meta-analysis has several limitations. First, the exclusion of non-English studies and those with incomplete data may introduce selection bias. Second, the variability in ERAS protocol components across studies limits direct comparability. Standardizing key components, such as nutritional interventions and postoperative monitoring, may significantly reduce variability and improve outcome generalizability. Finally, the absence of detailed cost-effectiveness analyses precludes a comprehensive evaluation of ERAS's economic impact, which is a critical consideration in healthcare decision-making. Cost-effectiveness evaluations could leverage existing administrative datasets or conduct micro-costing studies within randomized trials to provide actionable insights.

The findings support the widespread adoption of ERAS protocols in gastrointestinal and cardiovascular surgeries to standardize care and optimize outcomes. However, future research should focus on adapting ERAS protocols for non-surgical or outpatient settings to expand their utility, exploring pediatric and high-risk populations to address unique challenges like developmental needs or complex comorbidities, and leveraging unpublished data to enhance understanding in underexplored areas, such as cardiac surgeries or long-term outcomes.

## Conclusions

This study highlights the effectiveness of ERAS protocols in optimizing postoperative outcomes for gastrointestinal and cardiovascular surgeries. The findings have significant implications for updating surgical guidelines by incorporating ERAS as a standard approach, which could drive consistency in perioperative care practices. Additionally, they underscore the need for tailored resource allocation to ensure the availability of essential components, such as nutritional support and early mobilization interventions. ERAS protocols consistently reduced the LOS, minimized complications, and enhanced recovery across diverse patient populations and surgical contexts. The findings underscore the potential of ERAS to standardize care, promote faster rehabilitation, and improve perioperative management, particularly in urgent surgeries and patients with comorbidities. Actionable strategies, including the implementation of targeted training programs for surgical teams and pilot studies to evaluate ERAS in diverse healthcare settings, can facilitate the broader adoption and adaptation of ERAS protocols in routine practice. These results support the widespread adoption of ERAS as a cornerstone of modern surgical practice.

## References

[1] Baldus S, Doenst T, Pfister R (2024). Transcatheter repair versus mitral-valve surgery for secondary mitral regurgitation. N Engl J Med.

[2] Goldstone AB, Chiu P, Baiocchi M, Lingala B, Patrick WL, Fischbein MP, Woo YJ (2017). Mechanical or biologic prostheses for aortic-valve and mitral-valve replacement. N Engl J Med.

[3] Malvindi PG, Bifulco O, Berretta P (2024). The enhanced recovery after surgery approach in heart valve surgery: a systematic review of clinical studies. J Clin Med.

[4] Kubitz JC, Schulte-Uentrop L, Zoellner C (2020). Establishment of an enhanced recovery after surgery protocol in minimally invasive heart valve surgery. PLoS One.

[5] Gebauer A, Konertz J, Petersen J (2023). The impact of a standardized Enhanced Recovery After Surgery (ERAS) protocol in patients undergoing minimally invasive heart valve surgery. PLoS One.

[6] Baxter R, Squiers J, Conner W, Kent M, Fann J, Lobdell K, DiMaio JM (2020). Enhanced recovery after surgery: a narrative review of its application in cardiac surgery. Ann Thorac Surg.

[7] Yazdchi F, Hirji S, Harloff M (2022). Enhanced recovery after cardiac surgery: a propensity-matched analysis. Semin Thorac Cardiovasc Surg.

[8] Shida D, Tagawa K, Inada K (2017). Modified enhanced recovery after surgery (ERAS) protocols for patients with obstructive colorectal cancer. BMC Surg.

[9] Tanious MK, Ljungqvist O, Urman RD (2017). Enhanced recovery after surgery: history, evolution, guidelines, and future directions. Int Anesthesiol Clin.

[10] Emelinda BN, Amengle LA, Bengono RSB, Arlette MMJ, Ngongheh BA, Minkande JZ (2024). Practice of enhanced recovery after caesarean delivery: a randomised controlled clinical trial in a tertiary hospital in Yaoundé-Cameroon. J Obstet Anaesth Crit Care.

[11] Engelman DT, Ben Ali W, Williams JB (2019). Guidelines for perioperative care in cardiac surgery: enhanced recovery after surgery society recommendations. JAMA Surg.

[12] Maj G, Regesta T, Campanella A, Cavozza C, Parodi G, Audo A (2022). Optimal management of patients treated with minimally invasive cardiac surgery in the era of enhanced recovery after surgery and fast-track protocols: a narrative review. J Cardiothorac Vasc Anesth.

[13] McCarthy C, Fletcher N (2020). Early extubation in enhanced recovery from cardiac surgery. Crit Care Clin.

[14] Pędziwiatr M, Mavrikis J, Witowski J, Adamos A, Major P, Nowakowski M, Budzyński A (2018). Current status of enhanced recovery after surgery (ERAS) protocol in gastrointestinal surgery. Med Oncol.

[15] Feldheiser A, Aziz O, Baldini G (2016). Enhanced Recovery After Surgery (ERAS) for gastrointestinal surgery, part 2: consensus statement for anaesthesia practice. Acta Anaesthesiol Scand.

[16] Brooks NA, Kokorovic A, McGrath JS (2022). Critical analysis of quality of life and cost-effectiveness of enhanced recovery after surgery (ERAS) for patient's undergoing urologic oncology surgery: a systematic review. World J Urol.

[17] Shoucair S, Alnajjar S, Sattari A, Almanzar A, Lisle D, Gupta VK (2024). Impact of surgical resident education and EMR standardization in enhancing ERAS adherence and outcomes in colorectal surgery. J Surg Educ.

[18] Gaiwal S, Palep JH, Mirkute R, Prasad N, Kush M (2024). Low-pressure pneumoperitoneum with intraoperative dexmedetomidine infusion in laparoscopic cholecystectomy for enhanced recovery after surgery: a prospective randomised controlled clinical trial. J Minim Access Surg.

[19] Han L, Zhou Y, Wang Y (2025). Nutritional status of early oral feeding for gastric cancer patients after laparoscopic total gastrectomy: a retrospective cohort study. Eur J Surg Oncol.

[20] Haywood N, Mehaffey JH, Hawkins RB (2020). Gastrointestinal complications after cardiac surgery: highly morbid but improving over time. J Surg Res.

[21] Aykut A, Salman N, Demir ZA, Özgök A, Günaydın S (2024). Comparison of propofol and sevoflurane anaesthesia in terms of postoperative nausea-vomiting complication in cardiac surgery patients undergoing enhanced recovery after surgery protocol: a prospective randomized study. Turk J Anaesthesiol Reanim.

[22] Pasin L, Nardelli P, Landoni G (2019). Enhanced recovery after surgery program in elective infrarenal abdominal aortic aneurysm repair. J Cardiovasc Surg (Torino).

[23] Yang Q, Wang L, Zhang X (2024). Impact of an enhanced recovery after surgery program integrating cardiopulmonary rehabilitation on post-operative prognosis of patients treated with CABG: protocol of the ERAS-CaRe randomized controlled trial. BMC Pulm Med.

[24] Ashok A, Niyogi D, Ranganathan P (2020). The enhanced recovery after surgery (ERAS) protocol to promote recovery following esophageal cancer resection. Surg Today.

[25] Lassen K, Soop M, Nygren J (2009). Consensus review of optimal perioperative care in colorectal surgery: Enhanced Recovery After Surgery (ERAS) Group recommendations. Arch Surg.

[26] Ljungqvist O (2020). Enhanced Recovery After Surgery: a paradigm shift in perioperative care. Enhanced Recovery After Surgery.

[27] Lee Y, Yu J, Doumouras AG, Li J, Hong D (2020). Enhanced recovery after surgery (ERAS) versus standard recovery for elective gastric cancer surgery: a meta-analysis of randomized controlled trials. Surg Oncol.

[28] Nygren J, Thacker J, Carli F (2012). Guidelines for perioperative care in elective rectal/pelvic surgery: Enhanced Recovery After Surgery (ERAS®) Society recommendations. Clin Nutr.

[29] Tan P, Huo M, Zhou X, Zhao B (2023). The safety and effectiveness of enhanced recovery after surgery (ERAS) in older patients undergoing orthopedic surgery: a systematic review and meta-analysis. Arch Orthop Trauma Surg.

[30] O'Neill AM, Calpin GG, Norris L, Beirne JP (2023). The impact of enhanced recovery after gynaecological surgery: a systematic review and meta-analysis. Gynecol Oncol.

